# PD‐1/L1 inhibitors can improve but not replace chemotherapy for advanced urothelial carcinoma: A systematic review and network meta‐analysis

**DOI:** 10.1002/cai2.75

**Published:** 2023-05-19

**Authors:** Longkun Mao, Meihua Yang, Xinxiang Fan, Wenjie Li, Xiaodong Huang, Wang He, Tianxin Lin, Jian Huang

**Affiliations:** ^1^ Guangdong Provincial Key Laboratory of Malignant Tumor Epigenetics and Gene Regulation, Medical Research Center, Sun Yat‐Sen Memorial Hospital Sun Yat‐Sen (Zhongshan) University Guangzhou China; ^2^ Department of Urology, Sun Yat‐sen Memorial Hospital Sun Yat‐sen (Zhongshan) University Guangzhou China; ^3^ Guangdong Provincial Clinical Research Center for Urological Diseases Guangzhou China; ^4^ The First Affiliated Hospital of Nanchang University Nanchang China

**Keywords:** chemotherapy, efficacy, immune checkpoint inhibitors, safety, urinary bladder neoplasms

## Abstract

**Background:**

Programmed cell death‐1/ligand 1 inhibitors are a new treatment strategy for advanced urothelial carcinoma. Therefore, a comparative evaluation of their efficacy and toxicity compared with chemotherapy is necessary.

**Methods:**

We comprehensively searched PubMed, Web of Science, Embase, and Cochrane Library databases and performed a meta‐analysis of randomized controlled trials up to July 2021. We considered overall survival as the primary outcome, and progression‐free survival, objective response rate, and treatment‐related adverse events as secondary outcomes.

**Results:**

Overall, 3584 patients from five studies were evaluated. Compared with first‐line chemotherapy, programmed cell death‐1/ligand 1 inhibitors were significantly associated with worse progression‐free survival (*p* < 0.001) and adverse objective response rates (*p* < 0.001). However, the treatments were not significantly different in terms of overall survival (*p* = 0.33). Compared with second‐line chemotherapy, programmed cell death‐1/ligand 1 inhibitors significantly improved overall survival (*p* < 0.001), and there was no statistically significant difference in progression‐free survival (*p* = 0.89) or objective response rate (*p* = 0.34). Compared with chemotherapy, programmed cell death‐1/ligand 1 inhibitors were well tolerated (first‐line chemotherapy: *p* < 0.001; second‐line chemotherapy: *p* < 0.001).

**Conclusions:**

The efficacy of programmed cell death‐1/ligand 1 inhibitors in patients with advanced urothelial carcinoma is not superior to that of first‐line platinum‐based chemotherapy but is better than second‐line chemotherapy; however, programmed cell death‐1/ligand 1 inhibitors are safer than first‐ and second‐line chemotherapy and have a broader prospect for use in combination therapy.

## Graphical abstract

The efficacy of programmed cell death‐1/ligand 1 inhibitors in patients with advanced urothelial carcinoma is approximate to first‐line platinum‐based chemotherapy, and programmed cell death‐1/ligand 1 inhibitors are safer than first‐ and second‐line chemotherapy and have a broader prospect for use in combination therapy.

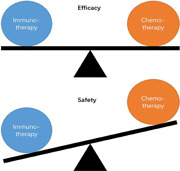

**Abbreviations**
CI: confidence interval
CTLA‐4: cytotoxic T‐lymphocyte‐associated protein 4
EV: enfortumab vedotin
FDA: Food and Drug Administration
FGFR: fibroblast growth factor receptor
GC: gemcitabine plus cisplatin
OR: odds ratio
ORR: objective response rate
OS: overall survival
PD‐1/L1: Programmed cell death‐1/ligand 1
PFS: progression‐free survival
RCTs: randomized clinical trials
TMB: tumor mutational burden
TRAEs: treatment‐related adverse events
UC: urothelial cancer
VEGF: vascular endothelial growth factor

## 1 INTRODUCTION

In 2020, there were 573,278 and 212,536 new cases of, and deaths from, bladder cancer, respectively [1], of which approximately 90% were urothelial carcinoma (UC) [2]. The 5‐year survival rate of locally advanced or metastatic UC is only 6.4% [3]. However, limited treatments are available for advanced UC, especially patients with unresectable locally advanced disease who can only receive chemotherapy.

Cisplatin has been considered the most effective cytotoxic treatment for advanced UC since 1980 [4]. Platinum‐based chemotherapy is now the first‐line treatment for advanced UC. Although chemotherapy plays a pivotal role in UC treatment, its development has plateaued. Over the past four decades, various doses, schedules, and sequences of platinum‐based regimens have been evaluated, even for platinum‐free double line or newer agent combinations. However, none of the new therapies have been an improvement on standard chemotherapy regimens [5]. Furthermore, the therapeutic effect of second‐line chemotherapy drugs is unsatisfactory, with the median overall survival (OS) afforded by vinflunine, paclitaxel, and docetaxel being only 5–7 months, and less than 10% of patients achieving an objective response [6]. Thus, to prolong survival in patients with advanced UC, new therapeutic schedules are necessary.

Immunotherapy has rapidly advanced recently and is now widely used in cancer treatment. The US Food and Drug Administration (FDA) has approved five programmed cell death‐1/ligand 1 (PD‐1/L1) inhibitors (atezolizumab, pembrolizumab, nivolumab, durvalumab, and avelumab) for patients with advanced UC in whom platinum‐based chemotherapy has failed. Furthermore, atezolizumab and pembrolizumab have been approved as first‐line treatments for patients ineligible for platinum‐based therapy [7]. However, the results of some recent randomized clinical trials (RCTs) on the efficacy and safety of PD‐1/L1 inhibitors found contradictory results. Therefore, we conducted a systematic review and meta‐analysis to clarify the efficacy and safety of PD‐1/L1 inhibitors compared with those of first‐ and second‐line chemotherapy to guide clinical practice.

## 2 EVIDENCE ACQUISITION

### 2.1 Literature search

Two authors (Longkun Mao and Meihua Yang) independently retrieved literature published by December 31, 2022 from PubMed, Cochrane Library, Embase, and Web of Science Core Collection without regional restrictions. The search was performed to identify all articles on the use of PD‐1/L1 inhibitors for treating advanced UC. The following terms were included in the search (all fields): (immunotherapy OR pembrolizumab OR nivolumab OR atezolizumab OR avelumab OR durvalumab) AND (advanced UC OR metastatic UC). The related article function was also used to expand the search scope. The computer search supplemented a manual search of the reference lists of all retrieved research, review articles, and conference abstracts.

### 2.2 Study eligibility

The inclusion criteria were as follows: (a) studies on advanced UC patients treated with a PD‐1/L1 inhibitor; (b) RCTs; (c) studies that reported data on median OS or progression‐free survival (PFS) or objective response rate (ORR) and treatment‐related adverse events (TRAEs); (d) studies containing at least two arms of the chemotherapy and immunotherapy groups; and (e) publications in English. Studies that did not meet the inclusion criteria were excluded. When more than one publication reported on the same study population, studies with the most updated and/or comprehensive data were included.

### 2.3 Data extraction

Data extraction and assessment were performed independently by two different investigators (Longkun Mao and Meihua Yang), and disagreements were resolved through consensus or by discussion with a third author (Xinxiang Fan). The following information was extracted from each study: (a) first author and publication year, study phase, study name, and clinical trial number; (b) whether neoadjuvant therapy was used, whether the patients were eligible to receive platinum‐based therapy, name and dose of the PD‐1/L1 inhibitors used, and name of the chemotherapeutic drugs used; (c) number of patients who received each treatment, and median follow‐up duration, median OS, median PFS, and ORR in the intention‐to‐treat population; and (d) numbers of TRAEs, numbers of patients with TRAEs of different grades, and the most common TRAEs.

### 2.4 Quality assessment

Two authors (Mao and Yang) independently assessed the quality of all included studies using RevMan 5.3 (Cochrane Collaboration), while a third author (Fan) addressed disagreements by consultation. The Cochrane Collaboration tool was used to assess the risk of bias, including sequence generation, incomplete outcome data, allocation concealment, selective outcome reporting, blinding of participants, personnel and outcome assessors, and other sources of bias.

### 2.5 Statistical analysis

The meta‐analysis endpoints were median OS, median PFS, ORR, and TRAEs. Forest plots were used for the summary variables of survival data and dichotomous outcomes, and all results were reported with 95% confidence intervals (CIs). The two‐side *Q* statistic and *I* ^2^ test were used to assess statistical heterogeneity between different studies; *p*‐values ≤ 0.05 were considered significant. The fixed‐effects model was chosen if there was no significant heterogeneity between studies; otherwise, the random‐effects model was used.

## 3 EVIDENCE SYNTHESIS

**Figure 1:**
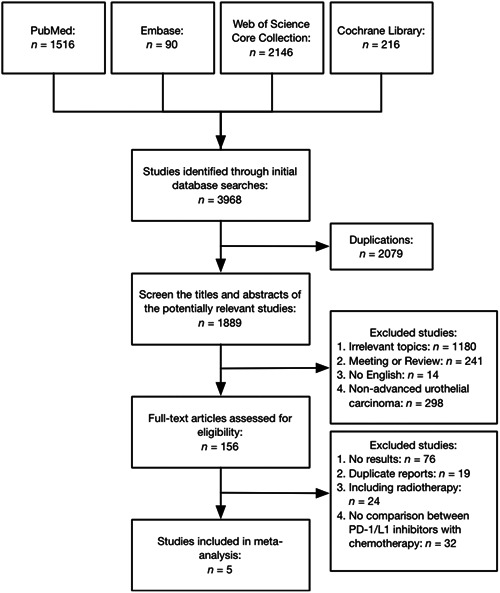
search flow diagram for this meta‐analysis.

We ultimately selected five eligible studies that included 3584 patients for analysis (Figure 1), of whom 1752 (48.9%) received PD‐1/L1 inhibitors [8, 9, 10, 11, 12]; hence, we included them in the meta‐analysis and divided them into three subgroups based on the PD‐1/L1 inhibitors used. Examination of these studies and the references listed in the review articles did not lead to any further evaluation studies. The agreement rates of the two reviewers regarding study selection and evaluation of trial quality were both 100%.

### 3.1 Characteristics of eligible studies

Characteristics of the included studies are listed in Table 1. The five meta‐analyses were divided into the following groups: immunotherapy compared with first‐line chemotherapy (cisplatin or carboplatin combined with gemcitabine) and immunotherapy compared with second‐line chemotherapy (paclitaxel, docetaxel, or vinflunine). The first‐line chemotherapy group contained three studies and included 2159 patients eligible for platinum‐based chemotherapy. Only two studies were in the second‐line chemotherapy group, with a total of 1473 patients who had shown progression after platinum chemotherapy or were ineligible for platinum‐based chemotherapy. The outcomes of all included studies are shown in Table 2.

**Table 1:** Characteristics of the included studies.

Author, year	PD‐1/L1 inhibitor	Chemo	Study name	Phase	NCT number	Study design	Line of therapy	Patients number		Eligible for cisplatin
								T	C	
Bellmunt [8]	Pembrolizumab	PDV	KEYNOTE‐045	3	NCT02256436	RCT	Previously treated	270	272	No
Powles [12]	Durvalumab	GC or GCa	DANUBE	3	NCT02516241	RCT	First line	346	344	Yes
Galsky [9]	Atezolizumab	GC or GCa	IMvigor130	3	NCT02807636	RCT	First line	362	400	Yes
Powles [11]	Atezolizumab	PDV	IMvigor211	3	NCT02302807	RCT	Previously treated	467	464	No
Powles [10]	Pembrolizumab	GC or GCa	KEYNOTE‐361 trial	3	NCT02853305	RCT	First line	307	352	Yes

Abbreviations: C, control; Chemo, chemotherapy; GC, gemcitabine plus cisplatin; GCa, gemcitabine plus carboplatin; PDV, paclitaxel, docetaxel, or vinflunine; RCT, randomized controlled trial; T, treatment.

**Table 2:** The outcomes of including studies.

Author, year	Median follow‐up	Median PFS (95% CI)		Median OS (95% CI)		ORR (95% CI)		Grade 1–2 TRAEs^a^		Grade 3+ TRAEs^a^		Most common TRAEs (%)
		T	C	T	C	T	C	T	C	T	C	
Bellmunt^b^ [8]	14.1 months	2.1 months (2.3–3.5)	3.3 months (2.3–3.5)	10.3 months (8–11.8)	7.4 months (6.1–8.3)	21.1% (16.4–26.5)	11.4% (7.9–15.8)	122 (45.9)	104 (40.8)	40 (15)	126 (49.4)	Pruritus (20)
Powles^c^ [12]	41.2 months	2.3 months (1.9–3.5)	6.7 months (5.7–7.3)	14.4 months (10.4–17.3)	12.1 months (10.4–15)	26%	49%	144 (42)	93 (30)	49 (14)	189 (60)	Fatigue (13)
Galsky^c^ [9]	11.8 months	NA	NA	15.7 months (13.1–17.8)	13.4 months (12.0–15.2)	23%	44%	154 (44)	54 (14)	57 (15)	319 (82)	Fatigue (11)
Powles^b^ [11]	17.3 months	2.1 months (2.1–2.2)	4.0 months (3.4–4.2)	8.6 months	8.0 months	13.4% (10.5–16.9)	13.4% (10.5–16.9)	224 (49)	197 (45)	95 (21)	198 (45)	Fatigue (15)
Powles^c^ [10]	31.7 months	NA	7.1 months (6.4–7.9)	15.6 months (12.1–17.9)	14.3 months (12.3–16.7)	30.3% (25.2–35.8)	44.9% (39.6–50.2)	135 (44.7)	7 (2)	57 (19)	315 (92)	Anemia (28)

Abbreviations: C, control; CI, confidence interval; Mo, months; NA, data not available; OS, overall survival; PFS, progression‐free survival; T, treatment; TRAEs, treatment‐related adverse events.

^a^ Data are *n* (%).

^b^ PD‐1/L1 inhibitors versus second‐line chemotherapy.

^c^ PD‐1/L1 inhibitors versus first‐line chemotherapy.

### 3.2 Quality assessment of the included studies

The risk of bias in the analyzed RCTs was evaluated and summarized in Appendix 1. Generally, a low risk of bias was identified, which met the general requirement for meta‐analyses.

### 3.3 Overall survival

**Figure 2:**
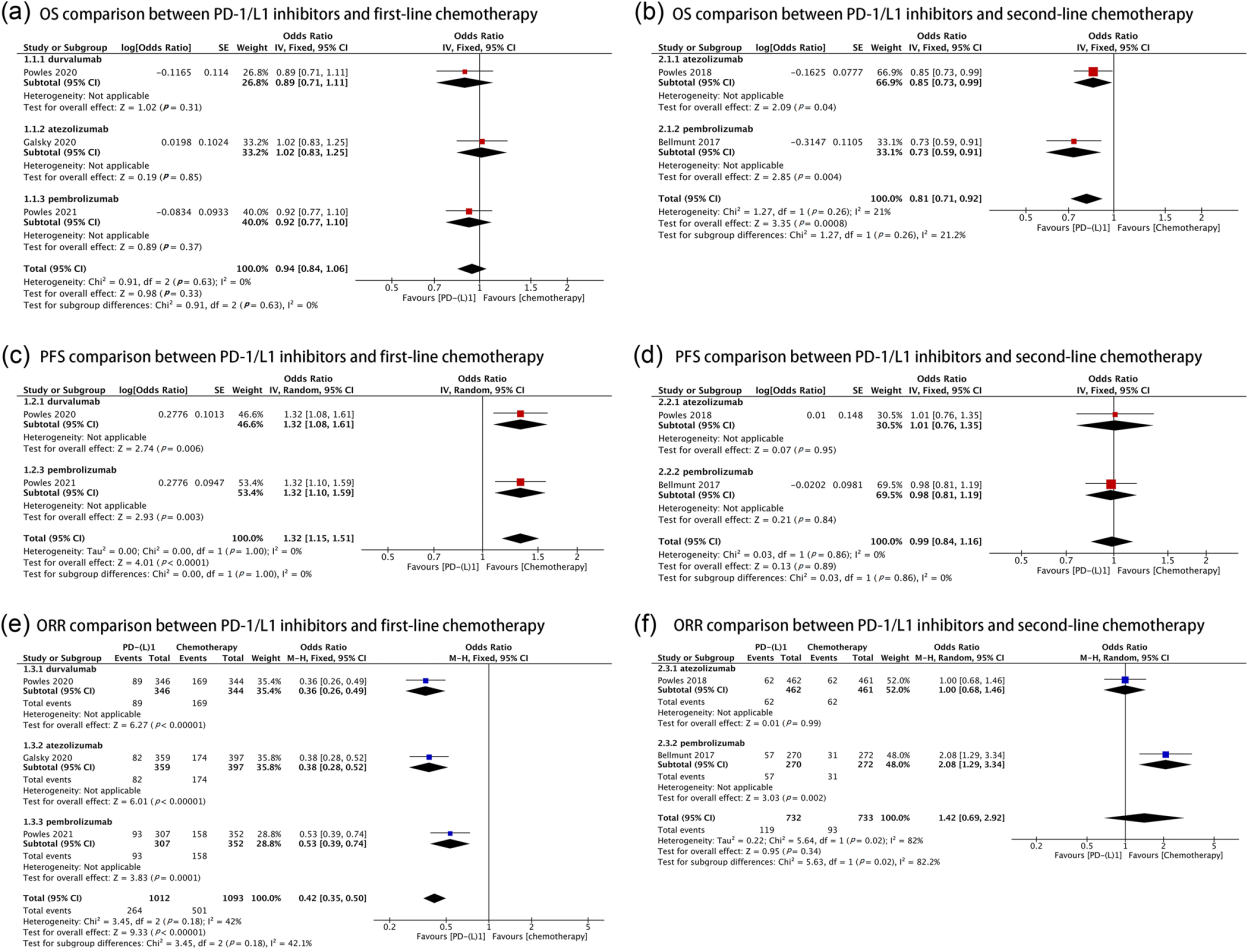
Forest plots of overall survival (OS), progression‐free survival (PFS), and objective response rate (ORR) in the intention‐to‐treat population comparing PD‐1/L1 inhibitors to chemotherapy. (a) OS comparison between PD‐1/L1 inhibitors and first‐line chemotherapy. (b) OS comparison between PD‐1/L1 inhibitors and second‐line chemotherapy. (c) PFS comparison between PD‐1/L1 inhibitors and first‐line chemotherapy. (d) PFS comparison between PD‐1/L1 inhibitors and second‐line chemotherapy. (e) ORR comparison between PD‐1/L1 inhibitors and first‐line chemotherapy. (f) ORR comparison between PD‐1/L1 inhibitors and second‐line chemotherapy.

Data were pooled from five studies (3584 patients) that evaluated OS. The results showed no statistically significant difference between immunotherapy and first‐line chemotherapy (odds ratio [OR] = 0.94, 95% CI: 0.84–1.06, *p* = 0.33, *I* ^2^ = 0%; Figure 2a). Furthermore, analysis of the durvalumab (OR = 0.89, 95% CI: 0.71–1.11, *p* = 0.31), atezolizumab (OR = 1.02, 95% CI: 0.83–1.25, *p* = 0.85), and pembrolizumab (OR = 0.92, 95% CI: 0.77–1.10, *p* = 0.37) subgroups revealed no statistically significant differences. In contrast, immunotherapy significantly improved OS compared with second‐line chemotherapy (OR = 0.81, 95% CI: 0.71–0.92, *p* < 0.001, *I* ^2^ = 21%; Figure 2b). Both the pembrolizumab (OR = 0.73, 95% CI: 0.59 to 0.91, *p* = 0.004) and atezolizumab (OR = 0.85, 95% CI: 0.73–0.99, *p* = 0.04) subgroups were associated with significant results.

### 3.4 Progression‐free survival

PFS data was derived from four studies, with two RCTs each in the first‐ and second‐line chemotherapy groups (1349 and 1473 patients, respectively). PD‐1/L1 inhibitors resulted in a significantly lower PFS than first‐line chemotherapy (OR = 1.32, 95% CI: 1.15–1.51, *p* < 0.001, *I* ^2^ = 0%; Figure 2c), regardless of whether durvalumab or pembrolizumab was used (*p* = 0.006 and *p* = 0.003, respectively). In contrast, no statistically significant difference in PFS was noted when comparing PD‐1/L1 inhibitors with second‐line chemotherapy (OR = 0.99, 95% CI: 0.84–1.16, *p* = 0.89, *I* ^2^ = 0%; Figure 2d). This result was the same as that of the subgroup analysis (atezolizumab [*p* = 0.95] and pembrolizumab [*p* = 0.8]).

### 3.5 Objective response rate

Three studies (2105 patients) in the first‐line chemotherapy group and two (1465 patients) in the second‐line chemotherapy group were included in the analysis of ORR. The pooled ORR of PD‐1/L1 inhibitors and first‐line chemotherapy was 26.1% versus 45.8%, respectively (OR = 0.42, 95% CI: 0.35–0.50, *p* < 0.001, *I* ^2^ = 42%; Figure 2e), AND additional subgroup analysis demonstrated that each PD‐1/L1 inhibitor had a significantly lower ORR than first‐line chemotherapy (*p* < 0.001 for each subgroup). In contrast, the pooled ORR of PD‐1/L1 inhibitors and second‐line chemotherapy was 16.3% versus 12.4%, respectively (OR = 1.42, 95% CI: 0.69–2.92, *p* = 0.34, *I* ^2^ = 82%; Figure 2f), and atezolizumab was not statistically different from second‐line chemotherapy, while pembrolizumab had a higher ORR (OR = 2.08, 95% CI: 1.29–3.34, *p* = 0.002).

### 3.6 Treatment‐related adverse events

All studies describing first‐line chemotherapy (three studies; 2053 patients) and second‐line chemotherapy (two studies; 1423 patients) reported TRAEs. The all‐grade TRAE occurrence rates for PD‐1/L1 inhibitors and first‐line chemotherapy were 68.7% and 94.5%, respectively, and the occurrence rates of grade 3/4+ TRAEs were 16.3% and 78.2%, respectively. For PD‐1/L1 inhibitors and second‐line chemotherapy, the all‐grade TRAEs occurrence rates were 66.3% and 89.5%, respectively, and occurrence rates for grade 3/4+ TRAEs were 18.6% and 46.4%, respectively.

**Figure 3:**
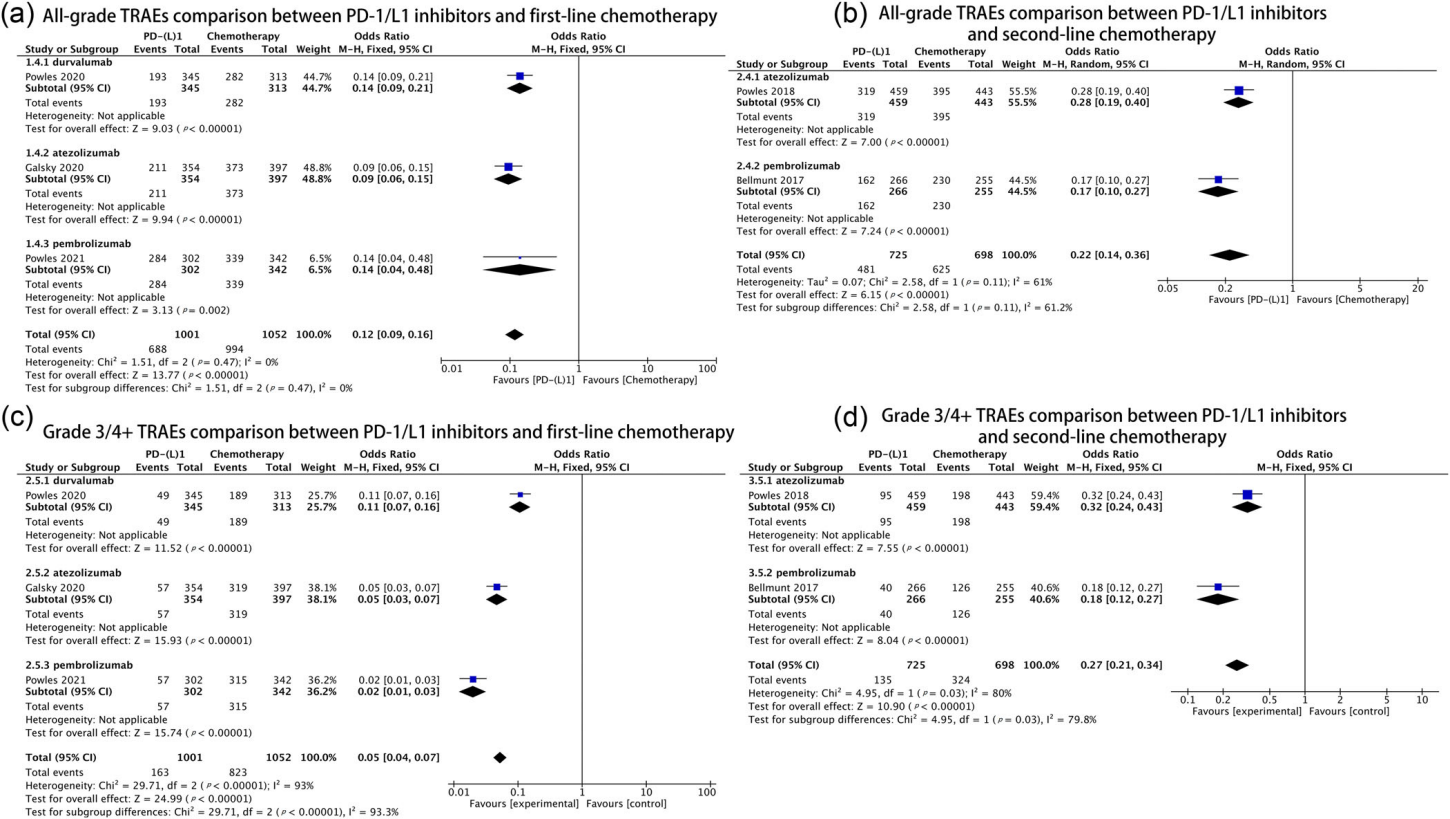
Forest plots of all‐grade and grade 3/4 + treatment‐related adverse events (TRAEs) in the intention‐to‐treat population comparing PD‐1/L1 inhibitors to chemotherapy. (a) Comparison of all‐grade TRAEs between PD‐1/L1 inhibitors and first‐line chemotherapy. (b) Comparison of all‐grade TRAEs between PD‐1/L1 inhibitors and second‐line chemotherapy. (c) Comparison of grade 3/4+ TRAEs between PD‐1/L1 inhibitors and first‐line chemotherapy. (d) Comparison of grade 3/4+ TRAEs between PD‐1/L1 inhibitors and second‐line chemotherapy.

PD‐1/L1 inhibitors were associated with a significantly lower incidence of all‐grade TRAEs (first‐line chemotherapy: OR = 0.12, 95% CI: 0.09–0.16, *p* < 0.001, *I* ^2^ = 0%; second‐line chemotherapy: OR = 0.22, 95% CI: 0.14–0.36, *p* < 0.001, *I* ^2^ = 61%; Figure 3a,b) and grade 3/4+ TRAEs (first‐line chemotherapy: OR = 0.05, 95% CI: 0.04–0.07, *p* < 0.001, *I* ^2^ = 93%; second‐line chemotherapy: OR = 0.27, 95% CI: 0.21–0.34, *p* = 0.03, *I* ^2^ = 80%; Figure 3c,d) compared with chemotherapy, and each subgroup showed similar results (*p* = 0.002 for pembrolizumab in the first‐line chemotherapy group for all‐grade TRAEs, *p* < 0.001 for others).

For patients receiving PD‐1/L1 inhibitors, the most common TRAEs were fatigue, pruritus, and anemia, whereas chemotherapy was associated with a greater risk of neutropenia and reductions in white blood cell and platelet counts.

## 4 DISCUSSION

This study is the first systematic meta‐analysis to explore the advantages and disadvantages of immunotherapy compared with first‐ and second‐line chemotherapy for advanced UC. This analysis included 3584 patients from five well‐organized RCTs and indirectly compared clinically relevant immunotherapy options. This approach generated several important findings: (a) although PD‐1/L1 inhibitors significantly reduced PFS and ORR compared with first‐line chemotherapy, they showed no statistically significant difference in OS; (b) PD‐1/L1 inhibitors significantly improved OS compared with second‐line chemotherapy, and showed no statistically significant difference in PFS and ORR; (c) PD‐1/L1 inhibitors were significantly better tolerated than first‐ and second‐line chemotherapy.

Under normal physiological conditions, the PD‐L1 pathway can prevent excessive immune responses and improve self‐tolerance by promoting antigen‐specific T‐cell apoptosis and inhibiting regulatory T‐cell apoptosis. However, this pathway can inhibit T‐cell survival, activate proliferation, and show cytotoxic effects in tumors. PD‐1/L1 inhibitors can relieve this inhibition and have achieved widespread and successful application since atezolizumab and pembrolizumab were approved by the FDA in 2017 for UC patients who were not suitable for cisplatin [7]. Our results showed that there was no statistically significant difference in OS between immunotherapy (8.6–15.7 months) and first‐line chemotherapy (7.4–14.3 months). For advanced UC patients who had progressed after a platinum‐containing regimen, the median OS after receiving PD‐1/L1 inhibitors was 8.6–10.3 months, which showed a significant survival benefit compared with second‐line chemotherapy (7.4–8.0 months).

Single‐agent immunotherapy has demonstrated OS benefits that were not superior to those obtained with first‐line chemotherapy in advanced UC patients, but the patients tolerated the treatments well, and the mechanism is complementary to that of other treatments. Therefore, researchers have felt that combination therapy is a viable future direction, with some trials of combination therapy achieving gratifying results.

The combination of cytotoxic chemotherapy and immunotherapy has been successfully used to treat cancer. For example, Galsky et al. [13] proved that gemcitabine plus cisplatin (GC) plus ipilimumab lead to a significant expansion of peripheral blood CD4 cells in metastatic UC patients, which is associated with improved survival. Recently, the KEYNOTE‐361 (NCT02853305) [10] and IMvigor 130 (NCT02807636) [9] trials reported that the addition of PD‐1/L1 inhibitors (pembrolizumab and atezolizumab, respectively) to first‐line chemotherapy could achieve improvements in OS (16–17 vs. 13.4–14.3 months) and PFS (8.2–8.3 vs. 6.3–7.1 months) compared with chemotherapy alone in advanced UC. Moreover, the rate of complete response to combination therapy was almost double that of chemotherapy, and the safety of combination therapy was similar to that of chemotherapy alone in the IMvigor 130 trial. However, PD‐1/L1 inhibitors plus chemotherapy did not show consistent benefits compared with chemotherapy alone, arguing for the current platinum‐based chemotherapy to remain the gold‐standard in the first‐line setting for the management of advanced UC.

In addition to cytotoxic drugs, the combination of targeted therapy with immunotherapy is favored by many researchers. Fibroblast growth factor receptor (FGFR) helps cells grow, survive, and multiply [14]. FGFR inhibitors such as erdafitinib, infigratinib, and rogaratinib have been proven to be effective in previous clinical trials (median OS of patients treated with erdafitinib and infigratinib was 13.8 months and 7.8 months, respectively, while the OR of patients treated with rogaratinib has not been reported), and erdafitinib was recently approved by the FDA for use in patients with metastatic UC who progressed on platinum‐based chemotherapy [15]. FGFR inhibitors are undoubtedly becoming a valuable part of the treatment for advanced bladder cancer. Currently, many clinical trials are being conducted to explore how best to combine FGFR inhibitors with PD‐1/L1 inhibitors to bring more survival benefits to patients [16]. Another common target is nectin‐4, a type 1 transmembrane protein that belongs to a family of cell adhesion molecules that are highly expressed in a variety of cancers, particularly in UC. Enfortumab vedotin (EV, also known as ASG‐22CE) is a monoclonal antibody‐drug conjugate (ADC) that selectively binds to nectin‐4‐expressing cells, results in their apoptotic death [17]. The single‐agent efficacy of EV in patients with metastatic UC had been confirmed in two early phase 1 clinical trials [18, 19] (ORR: 40%–33%, PFS: 17–23.1 weeks, respectively). These results prompted the FDA to accelerate the approval of EV for patients with advanced UC who showed disease progression after platinum‐based chemotherapy and immunotherapy in December 2019 [20]. Surprisingly, a study of EV with pembrolizumab in advanced UC (EV‐103) reported that the confirmed investigator‐assessed ORR was 73.3% [21]. Additionally, the ongoing EV‐302 study is comparing the efficacy and safety of this combination with GC chemotherapy.

Cytotoxic T‐lymphocyte‐associated protein 4 (CTLA‐4) is another immune checkpoint that has been shown to downregulate immune responses. Combinations of PD‐1/L1 and CTLA‐4 inhibitors such as nivolumab plus ipilimumab have been proven to show high ORR (25.6%–38.0%) in previously treated metastatic UC [22]. Additionally, an ongoing phase 3 trial [23] is expected to bring stronger evidence to support nivolumab plus ipilimumab becoming the first‐line treatment for metastatic UC. The combination of durvalumab plus tremelimumab was reported to demonstrate benefits in patients with high PD‐1/L1 expression in a phase 1 clinical trial [24]. Although a series of subsequent trials did not achieve the primary end points, durvalumab plus tremelimumab showed higher OS and ORR than durvalumab alone or chemotherapy [12, 25, 26, 27, 28].

Although immunotherapy has achieved much success, researchers continue to face many challenges regarding the ultimate goal of “curing cancer.” For example, only approximately a quarter of patients (ORR: 23%–30%) show benefits from immunotherapy, and some of them will ultimately face tumor progression (PFS: 1.9–3.5 months). This phenomenon is known as primary or secondary immune escape, and occurs when patients who have not previously received immunotherapy fail to respond to it or have stopped responding to immunotherapy after previously showing a response [29]. Primary immune escape may be associated with the suppression of T‐cell responses through multiple immunosuppressive mechanisms, which are related to differences in the immune environment at baseline. Mariathasan et al. [30] demonstrated that single‐agent immunotherapy performed poorly in immune‐excluded UC with elevated TGFβ signaling, and that combining anti‐TGFβ agents with immunotherapy could overcome primary immune escape. Additionally, for inflamed tumor mutational burden (TMB)‐low tumors, anti‐vascular endothelial growth factor (VEGF) therapy could overcome myeloid‐mediated immune suppression [31]. Nadal et al. [32] reported that cabozantinib (VEGF targeted therapy) plus nivolumab yielded a favorable ORR (50%) and median PFS (24.1 months) in the treatment of immunotherapy‐naïve patients with chemotherapy‐refractory metastatic UC; even in immunotherapy‐refractory patients, the ORR was 28%. The mechanisms of secondary escape may also be diverse but are generally associated with downregulation of immunogenic antigens and growth of cancer cell clones lacking immunogenic antigens. However, a lack of relevant tumor data has made studies on the drivers of secondary escape more challenging [29]. A better understanding of the different mechanisms of immune escape will facilitate the development of more targeted and effective treatments for different cancer types with distinct phenotypes at different stages.

Safety is another aspect of efficacy that is also important when evaluating a new type of treatment. Immunotherapy activates patients' own immune system, potentially affecting multiple organs throughout the body, especially the skin, gastrointestinal system, liver, and endocrine system. These adverse effects were usually grade 1/2 TRAEs, with serious adverse reactions only accounting for approximately 15% of TRAEs, and thus most patients could tolerate and continue immunotherapy, which was very different from the findings of cytotoxic chemotherapy. In other evaluations of the occurrence of serious adverse events, Weber et al. [33] observed that C‐reactive protein and interleukin‐6 levels above the median baseline values of these biomarkers were significantly associated with adverse events following immunotherapy. Additionally, a prospective cohort [34] study demonstrated that the predictive factors for grade 4/5 and fatal immunological toxicities were elevated neutrophil/lymphocyte ratio, performance status ≥2, and lung cancer, which could help identify patients at risk of severe adverse events. Compared with the cytotoxicity of chemotherapeutics, the effect of immunotherapy on normal cells in the patient's body was acceptable. Thus, despite the minor differences in efficacy, we were more inclined to choose safer immunotherapy as the first‐line treatment rather than chemotherapy. Finally, the safety of immunotherapy also provides a basis for combinations with other treatment modalities.

The main limitation of this study was that although all the included studies were RCTs, the number of studies was relatively small, and there was only one study in each subgroup. Second, the analyses of OS, PFS, ORR, and TRAEs included all patients in the intention‐to‐treat analysis, and no further analysis was performed on their biomarkers. Additionally, when we compared the most common TRAEs, we did not count the specific number of patients with each adverse reaction but roughly ranked the TRAEs according to the number of studies that reported the adverse reaction.

## 5 CONCLUSIONS

This meta‐analysis found that among patients with advanced UC, PD‐1/L1 inhibitors showed no superior efficacy but better safety than first‐line platinum‐based chemotherapy, offering broader prospects for combination therapy.

## AUTHOR CONTRIBUTIONS

**Longkun Mao**: Data curation (equal); writing—original draft (equal). **Meihua Yang**: Formal analysis (equal); writing—review and editing (equal). **Xinxiang Fan**: Conceptualization (equal); methodology (equal). **Wenjie Li**: Data curation (supporting); software (supporting). **Xiaodong Huang**: Resources (supporting); validation (supporting). **Wang He**: Investigation (lead); supervision (lead). **Tianxin Lin**: Project administration (lead); writing—review and editing (lead). **Jian Huang**: Conceptualization (lead); project administration (lead).

## CONFLICT OF INTEREST STATEMENT

The authors declare no conflict of interest.

## ETHICS STATEMENT

This paper is a research report that uses statistical methods to synthesize several independent clinical studies aiming at the same clinical problem for quantitative analysis. Therefore, the creation of this research article does not involve any formal investigation and research, nor does it involve human participation in research. Therefore, this article does not need IRB review.

## INFORMED CONSENT

Not applicable.

## Data Availability

The data used in the work is all open source data on the web.
